# A large-scale outbreak of hand, foot and mouth disease, France, as at 28 September 2021

**DOI:** 10.2807/1560-7917.ES.2021.26.43.2100978

**Published:** 2021-10-28

**Authors:** Audrey Mirand, Robert Cohen, Maxime Bisseux, Stéphanie Tomba, Fabienne Cahn Sellem, Nathalie Gelbert, Stéphane Béchet, Bruno Frandji, Christine Archimbaud, Amélie Brebion, Hélène Chabrolles, Christel Regagnon, Corinne Levy, Jean-Luc Bailly, Cécile Henquell

**Affiliations:** 1 CHU Clermont-Ferrand, Laboratoire de Virologie, Centre National de Référence des entérovirus et parechovirus - Laboratoire Associé, Clermont-Ferrand, France; 2 Université d’Auvergne, LMGE UMR CNRS 6023 Equipe EPIE - Epidémiologie et physiopathologie des infections à entérovirus, Faculté de Médecine, Clermont-Ferrand, France; 3 Association Clinique et Thérapeutique Infantile du Val de Marne (ACTIV), Créteil, France; 4 Association Française de Pédiatrie Ambulatoire (AFPA), Orléans, France; 5 CompuGroup Medical France, Nanterre, France

**Keywords:** enterovirus, paediatric infectious diseases surveillance, herpangina, hand, foot and mouth disease, syndromic surveillance, ambulatory network

## Abstract

We report a large-scale outbreak of hand, foot and mouth disease (HFMD) in France. As at 28 September 2021, 3,403 cases have been reported (47% higher than in 2018–19). We prospectively analysed 210 clinical samples; 190 (90.5%) were enterovirus-positive. Most children presented with atypical HFMD. Coxsackievirus (CV)A6 (49.5%; 94/190) was predominant; no enterovirus A71 was detected. Dermatological and neurological complications of HFMD justify prospective syndromic and virological surveillance for early detection of HFMD outbreaks and identification of associated types.

Hand, foot and mouth disease (HFMD) and herpangina (HA) are childhood diseases most commonly associated with various non-polio enterovirus (EV) types. HFMD/HA are usually benign, although neurological complications are often observed during large epidemics involving EV-A71 [1-3]. On 14 June 2021, the French Paediatric and Ambulatory Research in Infectious diseases (PARI) network informed the Associated Laboratory of the National Reference Centre (AL-NRC) for enteroviruses and parechoviruses of an unusual increase in HFMD/HA cases in children observed in calendar week 24. This alert triggered a virological investigation to detect whether the causative virus was an EV-A71 or a new EV variant. We aimed to describe the epidemiological, clinical and virological characteristics of this large-scale outbreak of HFMD/HA.

## Syndromic surveillance for hand, foot and mouth disease and herpangina

Since September 2017, PARI has been conducting prospective surveillance of the most common paediatric infectious diseases seen in outpatient settings. The EV-associated mucocutaneous illnesses HFMD/HA are included in the PARI surveillance panel [4]. The PARI network includes 113 paediatricians (as at September 2021; ca 4% of all French paediatricians) located throughout all French metropolitan regions except Corsica and is coordinated by the Association Clinique et Thérapeutique du Val de Marne (ACTIV). Each paediatrician uses the same medical software (AxiSanté 5–InfanSoft, CompuGroup Medical, Nanterre, France) for patient record management. The system enables paediatricians to report cases of HFMD/HA based on diagnoses according to the International Statistical Classification of Diseases, 10th revision, including B08.4 (enteroviral vesicular stomatitis with exanthem), B08.5 (enteroviral vesicular pharyngitis), B97.1 (enterovirus as the cause of diseases classified elsewhere), and R21 (rashes and other nonspecific skin eruptions suggesting enterovirus infection) [5]. Automated data extraction from computerised medical records tracks the clinical epidemiology of HFMD/HA. A weekly newsletter reporting the number of cases or each monitored infectious disease is sent to all paediatricians and the AL-NRC by ACTIV.

**Figure 1:**
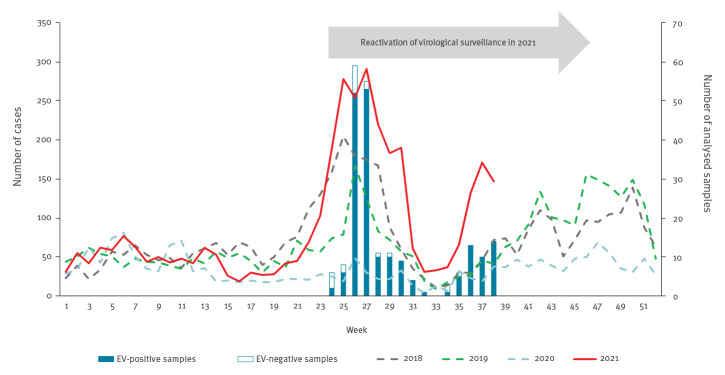
Weekly case numbers of hand, foot and mouth disease (HFMD) and herpangina (HA) reported by the Paediatric and Ambulatory Research in Infectious diseases network, 2018–2021 (n = 12,594) and of analysed clinical samples in children with HFMD/HA, France, 2021 (n = 210)

EV: enterovirus; HFMD: hand, foot and mouth disease; HA: herpangina; PARI: Paediatric and Ambulatory Research in Infectious diseases network.

A rapid increase in the number of mucocutaneous manifestations suggestive of EV infection in children was observed at week 24 in 2021 by the PARI network. The number of cases continued to increase until week 27, during which 291 cases were recorded (Figure 1). This is the highest number of weekly cases reported since syndromic surveillance began in 2017. A second HFMD/HA upsurge began in week 35 and peaked in week 37. As at week 38 in 2021, 3,403 cases were reported, compared to respective case numbers in 2020 (n = 1,201), 2019 (n = 1,996) and 2018 (n = 2,616) for the same period.

Vertical bar sections represent the number of EV-positive (blue bars) and -negative samples (white bars) since the reactivation of PARI virological surveillance for HFMD/HA at week 24 in 2021.

## Clinical and virological surveillance 

Syndromic surveillance was complemented by virological surveillance of HFMD/HA. Virological surveillance was first established in April 2014. After a suspension in 2020 because of the COVID pandemic, the surveillance was reactivated in 2021 during week 24, following the PARI network alert. Briefly, volunteer paediatricians in the PARI network collected, at discretion, throat or buccal swab specimens from children clinically diagnosed with HFMD/HA and sent samples with a standardised clinical report form to the AL-NRC for pan-EV testing (Enterovirus R-GENE, bioMérieux, Marcy-l’Étoile, France) and EV genotyping [6]. Based on the clinical items checked on the form, typical HFMD was defined by the presence of at least one typical localisation of HFMD, e.g. oral ulcerations, rash on palms, soles, buttocks, elbows or knees excluding any other site, whereas atypical HFMD was defined as HFMD with the presence of a rash at atypical sites or a generalised rash, or an atypical presentation such as Gianotti-Crosti syndrome (including papulo-vesicular eruption on buttocks and limbs) or eczema coxsackium. Herpangina was defined as localised oral ulcerations in the posterior part of the oral cavity [6]. 

**Figure 2:**
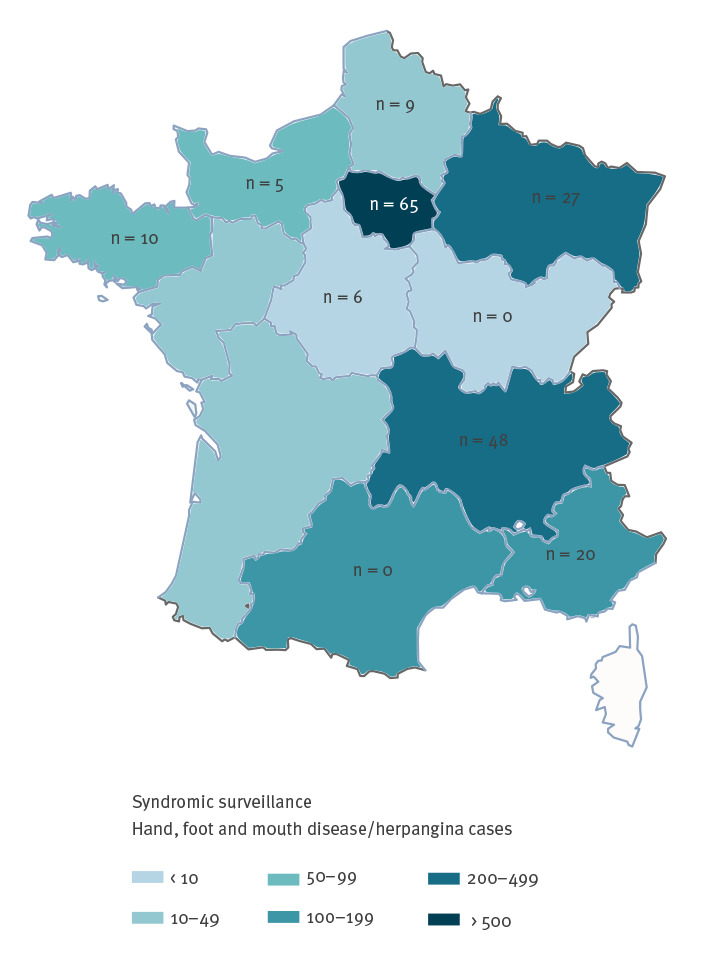
Geographical distribution of the number of hand, foot and mouth disease and herpangina cases and of enterovirus-positive samples in children, France, weeks 24–38, 2021 (n = 190)

As at 28 September 2021, 37.2% of paediatricians in the PARI network (42/113) across 10 of 13 French regions participated in clinical and virological surveillance. They prospectively sent 210 samples to the AL-NRC, which corresponded to 9.2% (210/2,283) of the cases reported by the PARI network between weeks 24 and 38. EV infection was confirmed in 90.5% (190/210) of children in eight regions (Figure 2). The mean age was 2.09 years (range: 21 days–7.4 years); the male:female sex ratio was 1.3. Fever or history of fever was reported in 78.9% (150/190) of EV-infected children. Most of them presented with atypical HFMD (64.2%; 122/190) associated with HA (30.5%; n = 58) or without HA (33.7%; n = 64); typical HFMD and HA alone were observed in 20.5% (n = 39) and 15.3% (n = 29) patients, respectively. Neurological signs including headache (n = 8), irritability (n = 31) and drowsiness (n = 5) were reported in 23.2% of children (n = 44) (Table 1).

Syndromic surveillance (colour gradient): the number of hand, foot and mouth disease and herpangina cases reported through the syndromic surveillance between weeks 24 and 38.

Virological surveillance (n per region): In each French metropolitan region, the n number indicates EV-positive samples. No samples were collected in five of the 13 regions, either because no paediatrician was involved in the virological surveillance in these regions (n = 3) or because no sample was collected (n = 2).

**Table 1:** Clinical features of children with enterovirus infections associated with hand, foot and mouth disease and herpangina, France, weeks 24–38 (n = 190)

Clinical characteristics	EV-infected children^a^ (n = 190)	
	n	%
**General signs of illness**		
Fever or history of fever	150	78.9
Asthenia	55	28.9
**Localisations of eruptions**		
Palms	100	52.6
Soles	104	54.7
Buttocks	98	51.6
Elbows/knees	50	26.3
Lower limbs	58	30.5
Upper limbs	45	23.7
Generalised eruption	14	7.4
Trunk	22	11.6
Face	68	35.8
Giannotti-Crosti syndrome^b^	12	6.3
Eczema coxsackium	11	5.8
**Diagnosis**		
Typical HFMD ± HA	39	20.5
Atypical HFMD ± HA	122	64.2
HA alone	29	15.3
**Other signs of illness**		
Digestive	64	33.7
Respiratory	80	42.1
Ear, nose, throat	56	29.5
Neurological	44	23.2

EV: enterovirus; HFMD: hand, foot and mouth disease; HA: herpangina.

^a^ Children with a laboratory-confirmed EV infection.

^b^ Giannotti-Crosti syndrome symptoms includes papulo-vesicular eruption on buttocks, face and limbs. The trunk, palms and soles are usually not affected.

**Figure 3:**
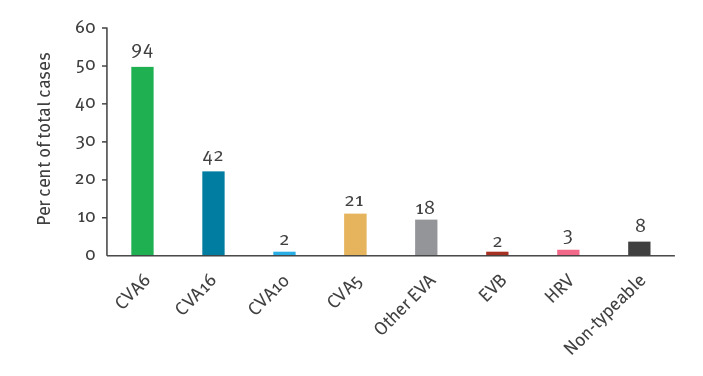
Distribution of enterovirus types associated with hand, foot and mouth disease and herpangina in children, France, weeks 24–38, 2021 (n = 190)

CV: coxsackievirus; EV: enterovirus; HRV: human rhinovirus.

Identification of the EV genotype was achieved for 95.8% (182/190) samples. EV-A71 was not detected. Another EV A type was identified in 93.2% (177/190) of children; coxsackievirus A6 (CVA6) was the most predominant type (n = 94; 49.5%) followed by CVA16 (n = 42; 22.1%) and CVA5 (n = 21; 11.1%) (Figure 3). CVA6 and CVA16 were mostly associated with atypical HFMD (75/94; 79.8% and 26/42; 61.9%, respectively) and CVA5 was more frequently associated with herpangina alone (11/21; 52.4%). 

Virological surveillance of enterovirus types from 8 French regions was performed. The n number above each bar indicates the number of each type from 190 EV-positive samples in children.

**Figure 4:**
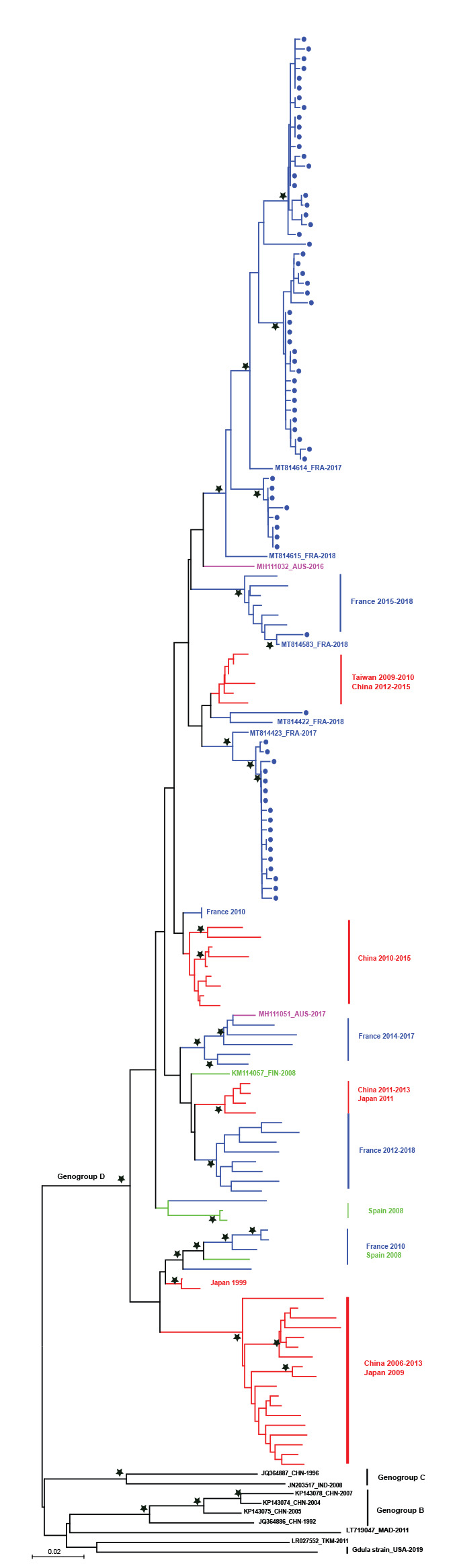
Phylogenetic tree of coxsackievirus A6 sequences from virological surveillance of hand, foot and mouth disease and herpangina in children, France, weeks 24–38, 2021 (n = 71)

The phylogenetic analysis of 71 partial VP1 sequences of the 94 CVA6 strains sampled in France in 2021 showed that 69 sequences were grouped in two main distinct lineages within the genogroup D. The two remaining sequences were dispersed within the genogroup D. All sequences displayed close genetic relationships with viruses recovered in France in 2017–18 (Figure 4). However, whole genome analysis of viral strains is needed to determine whether the strains involved in the 2021 epidemic could represent new variant forms. Partial VP1 sequences were deposited in GenBank (accession numbers: OK635614 to OK635790).

The phylogenetic tree was constructed by the neighbour-joining method and evaluated with 1,000 bootstrap pseudoreplicates, using Molecular Evolutionary Genetics Analysis (MEGA) version 6.0 software [19]. Only bootstrap values ≥ 70% are indicated by a black star. Genetic distances were calculated with Tamura-Nei’s model of evolution and branch length is drawn to the indicated scale (proportion of nt substitutions per site). Sequences were 495 bp long and started at nt 1 relative to the 1D gene of the Gdula prototype strain. The analysis was performed with 71 partial VP1 sequences of 94 CVA6 strains. The remaining 23 sequences were less than 495 bp long. Taxon names of all sequences are provided in Supplement. The strains collected in 2021 are labelled with a filled circle. Geographical origin and time of isolation of strains are indicated by the ISO-code abbreviation followed by the year of isolation. Genogroups were designed as previously described [20].

## Ethical statement

Informed consent was obtained from all parents or guardians of children. The syndromic surveillance was approved by the ethical committee of CHI Créteil Hospital, France [4]. The virological surveillance was approved by the review committee of the University Hospital of Clermont-Ferrand, France (reference AU1098).

## Discussion

The PARI network revealed a large-scale outbreak of HFMD/HA in France in 2021. The number of cases was 47% higher than in years 2018–19 during the same time period. Virological surveillance from week 24 showed that this epidemic was mainly associated with the CVA6 type. The biannual pattern in summer and autumn was similar to that usually expected for these diseases in Europe or Asia [2,4,7]. However, the 2021 epidemic was characterised by a sharp rise in the summer and an earlier autumn peak compared with previous years. This pattern may reflect that a higher proportion of children was susceptible to viral infections including EV. The year 2020 was, in fact, marked by a very low circulation of EV, both among hospitalised patients [8] and among children seen in primary care. In response to the COVID-19 pandemic, hygiene reinforcement may have reduced infectious contacts and immune stimulation, consequently leading to a greater susceptibility to infections in children [9]. A similar phenomenon was illustrated by the recently reported outbreak of respiratory syncytial virus (RSV) infections in Japan in July 2021 [10], which was substantially larger and earlier compared with previous years. The same situation could be observed in Europe for RSV infections [11]. 

In our study, we did not observe a shift in the age of disease onset; the mean age of children with HFMD/HA was similar to that of children in the 2014 epidemic (2.09 vs 2.10 years) [6]. Although we have not yet found available epidemiological data worldwide on HFMD/HA for the year 2021, it seems unlikely that the epidemic is limited to France. Signals of an HFMD/HA outbreak may have been missed elsewhere in Europe because most existing surveillance systems are hospital-based and focus on neurological EV infections [12]. 

We found that nearly two thirds of the children with confirmed EV-associated HFMD/HA in our study presented with atypical disease, a proportion usually observed in epidemics associated with CVA6 [6,13]. Since its re-emergence in 2008, this type has been commonly associated with atypical forms of the disease [6,13], sometimes affecting the whole body or associated with late-onset symptoms such as onychomadesis [14]. In addition, CVA16 was more frequently associated with atypical disease forms in 2021 than in 2014 (61.9%; n = 26/42 vs 34.1%; n = 42/123) [6]. Moreover, while paediatricians are very familiar with HMFD/HA, other clinicians should also be aware of these diseases, as they are not uncommon in adults living with children. In a CVA6-associated outbreak described in Finland in 2008, one third of the patients with HFMD/HA were adults [15]. As in children, presentation of the disease in adults can be atypical, which can make the diagnosis difficult and, in some cases, lead to hospitalisation [16]. 

In our primary care-based study, no data on hospitalisation were collected. As some HFMD/HA cases may be complicated by extensive skin manifestations or severe neurological conditions, this information would be valuable, as it could allow the close monitoring of hospitalised cases of HFMD/HA to detect a change in the clinical presentation or in the frequency of complications associated with these diseases. Data on hospitalised cases of HFMD/HA could be collected through hospital-based surveillance of EV infections as proposed by the European Non-Poliovirus Enterovirus Network (ENPEN), which has established standardised protocols for surveillance for HFMD, respiratory and neurological infections caused by EV and parechoviruses [17]. In France, no increase in the number of hospitalised cases of HFMD/HA has been observed through the EV hospital-based surveillance [8]. 

## Conclusions

The widespread circulation of non-polio EV, the epidemic pattern of EV infections and the recent emergence of EV-D68 and EV-A71 involved in severe neurological conditions warrant reinforcement of the surveillance of EV infections [18]. The PARI ambulatory syndromic surveillance proved to be effective for the early tracking of the 2021 outbreak. Detection of future HFMD/HA outbreaks caused by EV-A71 or an emergent EV could be achieved in combination with virological investigations. This study shows that a paediatric ambulatory network is of value for timely detection of diseases mostly seen within primary care settings.

## Supplementary Data

71000 Supplement
