# Supplementary material for: A large-scale outbreak of hand, foot and mouth disease, France, as at 28 September 2021
Source: Euro Surveill. 2021 Oct 28;26(43):2100978. doi: 10.2807/1560-7917.ES.2021.26.43.2100978 (PMC8555367; doi:10.2807/1560-7917.ES.2021.26.43.2100978)

This supplementary material is hosted by Eurosurveillance as supporting information alongside the article "**Large-scale outbreak of hand-foot and mouth disease, France, as at 28 September 2021**" on behalf of the authors who remain responsible for the accuracy and appropriateness of the content. the same standards for ethics, copyright, attributions and permissions as for the article apply.
Eurosurveillance is not responsible for the maintenance of any links or email addresses provided herein.

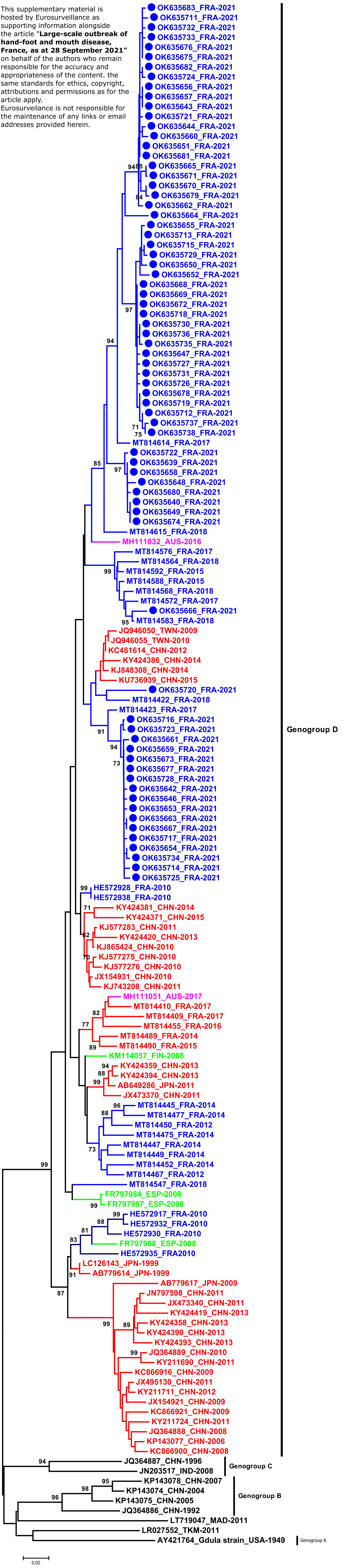

Supplement: Supplement [file 21-00978_MIRAND_Supplement.pdf]
